# Influence of calcium hydroxide/nano-chitosan as intracanal medication on push-out bond strength of epoxy resin and bioceramic sealers: an *ex-vivo* study

**DOI:** 10.1186/s12903-026-09219-0

**Published:** 2026-07-18

**Authors:** Khaled A. Ghozi, Mohamed A. Gomaa, Amany E. Badr

**Affiliations:** Department of Endodontics, Faculty of Dentistry, Mansoura University, Mansoura, 35516 Egypt

**Keywords:** AH Plus, Disinfection, Nanoparticles, NeoSEALER Flo, Universal testing machine

## Abstract

**Background:**

The present study aimed to evaluate the impact of intracanal medicaments—calcium hydroxide (CH) and calcium hydroxide/nano-chitosan (CH/NC)—on the push-out bond strength (POBS) of AH Plus (AHP) and NeoSEALER Flo (NSF).

**Methods:**

Nano-chitosan (NC) characterization was done using transmission electron microscopy (TEM), dynamic light scattering (DLS) and zeta potential analysis. Sixty extracted maxillary central incisors were instrumented up to X5 (50/0.06). Based on intracanal medicament used, specimens were allocated into three groups (*n* = 20): Group I; no intracanal medicament (control), Group II; (CH), and Group III; (CH/NC). Intracanal medicaments were removed after one week of incubation. Each group was subdivided based on sealer used (*n* = 10): subgroup A (AHP) and subgroup B (NSF). Obturation was performed with cold lateral compaction. After one week of incubation, roots were sectioned and a universal testing machine was used to evaluate POBS. Three-way ANOVA and Chi-square tests were conducted at 0.05 significance level.

**Results:**

TEM showed mostly spherical NC particles with diameters less than 50 nm. DLS analysis revealed a hydrodynamic Z-average diameter of 62.4 nm with a polydispersity index of 0.195. Zeta potential analysis demonstrated a positive surface charge of + 47.57 mV. The “medication” factor, “medication/sealer”, “medication/root level” and “sealer/root level” interactions significantly affected POBS (*P* < 0.05). Neither “sealer” nor “root level” factors significantly affected POBS (*P* > 0.05). Pooled POBS data revealed that: IA and IB subgroups were significantly higher than IIA, IIB, IIIA and IIIB subgroups (*P* < 0.05). Regarding failure mode, tested subgroups showed no significant difference (*P* > 0.05).

**Conclusions:**

Under the conditions of this study, prior application of CH or CH/NC reduced POBS for both sealers compared with controls. The addition of NC to CH did not provide a statistically significant improvement in POBS compared to CH alone. Overall, AHP and NSF demonstrated comparable POBS to root canal dentin.

## Background

Root canal treatment focuses on eradicating microorganisms and preventing reinfection by combining meticulous chemo-mechanical preparation, thorough intracanal disinfection, and a fluid-tight seal of the canal system [1–3]. Despite advancements in instrumentation and irrigant activation techniques, absolute eradication of microbes and debris is challenging because of the complex and variable anatomy of root canals. Consequently, intracanal medicaments are recommended as an adjunct during the interval between appointments to improve disinfection, decrease residual microbial presence, and enhance overall treatment success [1, 4, 5].

Calcium hydroxide (CH) is widely recognized as the gold standard because of its strong alkaline nature, extensive antibacterial properties, and capability to induce mineralization [2]. Sustained hydroxyl ion release contributes to the antibacterial action of CH as it disrupts bacterial membranes, denatures proteins, and damages DNA. Moreover, CH can neutralize bacterial endotoxins and stimulate hard tissue repair [2, 3]. Nevertheless, buffering effect of dentin and tissue remnants can neutralize hydroxyl ions, diminishing antimicrobial efficacy of CH. Additionally, activity of CH is reduced against resistant species such as *Enterococcus faecalis* (*E. faecalis)* and *Candida albicans*. Furthermore, complete removal of CH remnants from root canal system is challenging, and their persistence may impede sealer penetration into dentinal tubules, thereby affecting bond strength of sealer to root canal dentin. Prolonged use has also been associated with alterations in dentin properties, increasing susceptibility to root fractures [3]. These limitations have led to continued investigation of alternative intracanal medicaments.

Nanotechnology has enabled the development of nanomaterials sized 1 to 100 nm. Advances in nanotechnology have brought forth new approaches in root canal disinfection, leveraging the unique features of nanoparticles—such as their extremely small size, increased surface area and enhanced chemical reactivity. Nano-chitosan (NC), a naturally derived polysaccharide, possess unique properties that make them promising candidates for endodontic applications [4]. NC exhibits broad-spectrum antimicrobial activity, biocompatibility, and chelating ability, while its nanoscale dimensions facilitate deeper penetration into dentinal tubules and stronger interaction with microbial cells [6]. Incorporation of NC into CH pastes prolongs calcium ion release, sustains high pH levels, and enhances antimicrobial action against resistant pathogens such as *E. faecalis* [7].

Achieving a three-dimensional seal that is impermeable to fluids within the root canal system is a key goal of endodontic treatment. Gutta-percha (GP), the core material commonly used for obturation, does not adhere to canal walls. Therefore, root canal sealers are essential to fill voids and establish adhesion between dentin and core material. Achieving this seal is critical for preventing bacterial leakage and warranting long-term treatment success [8, 9].

Epoxy resin-based sealers (ERBSs) and calcium silicate-based sealers (CSBSs) are among the most widely used classes of root canal sealers in clinical practice, favored for their superior physicochemical properties, biocompatibility, and sealing ability. AH Plus (AHP), an ERBS, is widely used due to its favorable physicochemical properties and strong adhesion to dentin [10]. Recently, CSBSs, such as NeoSEALER Flo (NSF), have been developed. This bioactive sealer promotes hydroxyapatite formation, adheres to root dentin by means of both micromechanical interlocking and chemical bonding, and supports periapical healing [11, 12]. However, interaction between sealers and dentin may be significantly influenced by type of intracanal medicament used, as residual CH or its modified forms may affect adhesion [13, 14].

The rationale for combining NC with CH as a medicament partly rests on the potential of NC to modulate the dentinal substrate in a way that might preserve or improve sealer adhesion. This hypothesis was inspired by a previous study by Harishma et al. [15], who found that the addition of NC to an ERBS and a CSBS enhanced their POBS to root canal dentin compared to unmodified sealers.

Limited evidence is available on the impact of combining CH and NC as an intracanal medicament on push-out bond strength (POBS) of root canal sealers. Thus, this study aimed to assess the impact of calcium hydroxide/nano-chitosan (CH/NC) versus CH intracanal medicament on POBS of both ERBSs and CSBSs.

## Methods

### Ethical considerations

The Research Ethical Committee of Faculty of Dentistry, Mansoura University approved the protocol of this study (protocol ID: A0108024 RC).

**Fig. 1:**
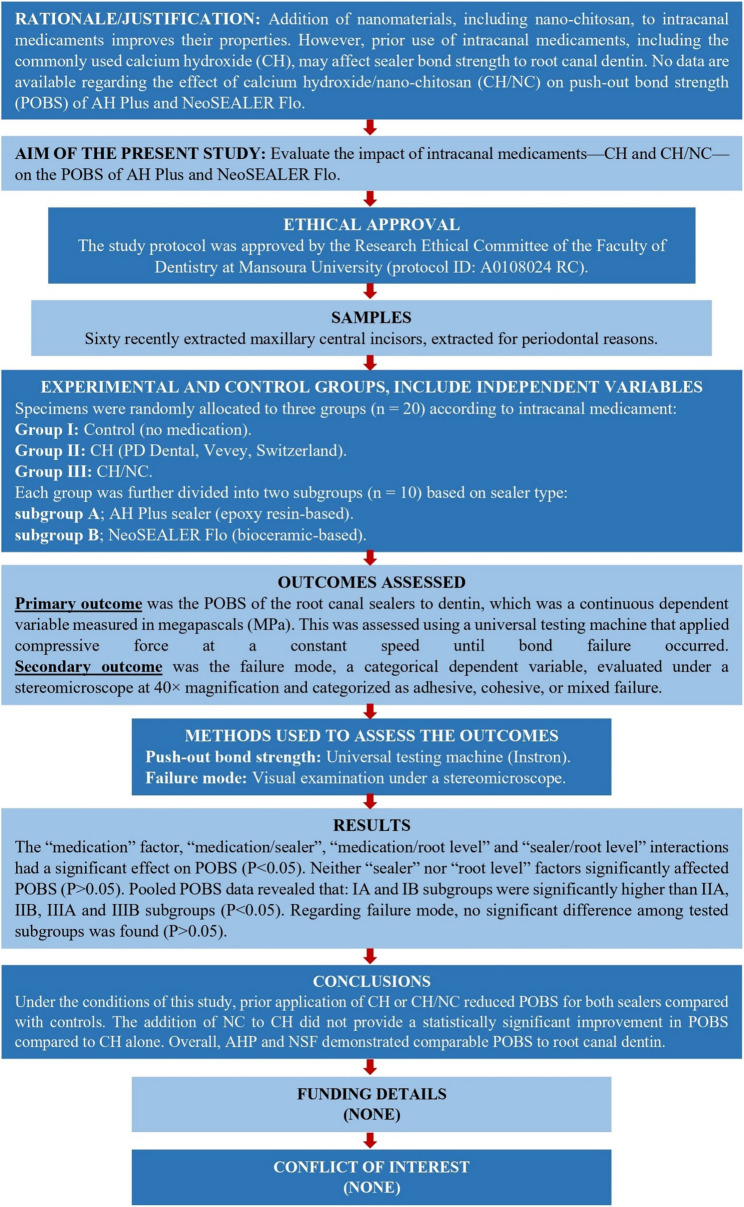
PRILE flowchart of the study

The present study followed the guidelines outlined in the Preferred Reporting Items for Laboratory Studies in Endodontology (PRILE) 2021 guidelines (Fig. 1) [16].

### Sample size calculation

Based on POBS data of a preceding study [5], sample size was calculated by G*Power 3.1 software (Heinrich Heine University, Düsseldorf, Germany) using ANOVA test at 80% power, alpha value (⍺) of 0.05 and effect size (f) of 0.554. A priori power analysis revealed that at least 48 teeth should be the total sample size. Consequently, the total sample size was increased to 60 teeth to increase the power, 10 teeth were used for each subgroup.

### Preparation of experimental materials

#### Preparation of nano-chitosan from chitosan

NC was synthesized via ionic gelation by dissolving 20 mg medium molecular weight chitosan (Oxford Lab Fine Chem LLP, Mumbai, India) in 40 mL of 2.0% (v/v) acetic acid. Then, 20 mL of 0.75 mg/mL aqueous sodium tripolyphosphate solution was added dropwise under stirring to induce ionic cross-linking, forming a NC suspension [17]. This suspension was freeze-dried to obtain a stable dry powder using an Alpha 1–2 LDplus freeze dryer (Martin Christ Gefriertrocknungsanlagen GmbH, Lower Saxony, Germany), where freezing was followed by vacuum sublimation to preserve nanoparticle structure and enhance stability. NC powder was kept in a sealed container at 4 °C, protected from light and moisture until use [18].

### Characterization methods of nano-chitosan

#### Transmission electron microscopy (TEM)

Powder of NC dissolved in glacial acetic acid was examined using JEOL JEM-2100 (JEOL Ltd., Tokyo, Japan) high-resolution transmission electron microscope, at an accelerating voltage of 200 kV, to assess morphology and size of NC.

#### Dynamic light scattering (DLS) and zeta potential measurement

The hydrodynamic diameter and surface charge of the NC were determined using a Malvern Zetasizer (Malvern Panalytical, Malvern, UK). The zeta potential measurement involved 12 Zeta runs over a period of 120 s, with a 10-second interval at ambient temperature.

#### Preparing 0.2% (w/v) nano-chitosan

A 0.3 N acetic acid solution was prepared by transferring about 1.8 mL glacial acetic acid (Advent Chembio Pvt. Ltd., Mumbai, India) into a 100-mL volumetric flask, then the volume was made up with distilled water. Subsequently, 20 mg of NC powder was accurately weighed on a M214Ai analytical balance (BEL Engineering, Monza, Italy) and added to the prepared acetic acid solution. The resulting mixture was vigorously stirred using the MSH-20 A magnetic stirrer (Daihan Scientific, Wonju, South Korea) until the powder was fully dissolved. NC solution was then stored at room temperature [4].

### Selection of teeth

Sixty freshly extracted maxillary central incisors with single, straight, mature roots and patent root canals were included. Teeth with cracks, resorption, caries, or prior root canal treatment were excluded. They were disinfected by immersion in 20 ml of 5.25% sodium hypochlorite (NaOCl). Then, they were mechanically cleaned with an ultrasonic scaler (Satelec Acteon group, Mérignac, France) to remove soft and hard tissue debris. A dental operating microscope (Leica Microsystems, Wetzlar, Germany) was used to verify the absence of cracks or external resorption. Preoperative mesiodistal and buccolingual radiographs were taken with NanoPix X-ray sensor (Eighteeth, Jiangsu Province, China) to check canal anatomy. Prior to use, the selected teeth were kept immersed in a 0.9% saline solution at room temperature.

### Preparation of specimens

Decoronation of teeth was done using a high-speed flat-ended diamond stone with water coolant to a standardized root length of 16 mm. Working length was set by inserting a size 10 K-file until visible at the apical foramen, subtracting 1 mm, and verified radiographically with a size 15 K-file. Canals were shaped using ProTaper Next rotary files (Dentsply Sirona, NC, USA) up to X5 (50/0.06) using circumferential brushing motion [19]. Patency was maintained with a size 15 K-file. After each file, canals were irrigated with 5 mL of 5.25% NaOCl via a 30-gauge side-vented needle (PD Dental, Vevey, Switzerland). Final irrigation included 5 mL distilled water, 5 mL 17% EDTA with ultrasonic activation for 1 min by Acteon Irrisafe Ultrasonic Tip (Satelec Acteon group, Mérignac, France), and 5 mL distilled water, with the needle inserted shorter than working length by 2 mm.

### Grouping of specimens

Specimens were randomly allocated into three groups (*n* = 20) based on the intracanal medication used as follows:

- Group I: No intracanal medicament (Control group).
- Group II: CH (PD Dental, Vevey, Switzerland) (CH group).
- Group III: CH/NC (CH/NC group).

### Application and removal of intracanal medicament

All canals were dried with size 50 paper points (Meta Biomed, Cheongju, Korea) until visually dry. For the CH group, a creamy paste was prepared by mixing CH powder with distilled water (1:1.5 w/v). For the CH/NC group, CH powder was mixed with freshly prepared 0.2% NC solution to achieve a final concentration of 100 mg CH per 1 mL of NC solution [4]. Medicaments were applied using a lentulo spiral (Dentsply Maillefer, Ballaigues, Switzerland) and visually confirmed. Canals were sealed coronally with 3 mm of Cavit (3 M ESPE, Seefeld, Germany). Specimens were wrapped in saline-soaked gauze to maintain 100% humidity and incubated at 37 °C for one week in a BTC incubator (Biotech, Giza, Egypt). After incubation, temporary fillings were removed, and all specimens were irrigated with 5 mL 17% EDTA activated by passive ultrasonic irrigation (PUI) with Acteon Irrisafe Ultrasonic Tip for 1 min, then rinsed with 5 mL distilled water.

### Obturation of specimens

Based on the sealer type used, each group was subdivided into two subgroups (*n* = 10) as follows:

- Subgroup A: AHP (Dentsply Sirona, Konstanz, Germany)
- Subgroup B: NSF (Avalon Biomed, TX, USA).

Canals were dried with size 50 paper points. Cold lateral compaction was the applied technique of obturation. Master GP cones size 50/0.04 (Meta Biomed, Cheongju, Korea), coated with sealer at their tips, were slowly inserted to the working length while lightly coating the canal walls with sealer. A size 30 finger spreader was used alongside the master cone, and accessory GP cones (25/0.02) were inserted sequentially until resistance to spreader penetration was encountered, typically requiring 4–6 accessory cones per canal. Radiographs confirmed void-free obturation before, and after, excess GP was removed. Coronal cavities were sealed with 3 mm of Cavit, wrapped in saline-soaked gauze, and incubated at 37 °C for one week.

### Push-out bond strength analysis

Each root was vertically embedded in chemically cured acrylic resin. Most coronal 3 mm and apical 2 mm of each root were sectioned and discarded using a water-cooled IsoMet 4000 microsaw (Buehler, Illinois, USA) equipped with a 0.6-mm thick diamond disc. Three 2-mm-thick slices were obtained from apical, middle, and coronal levels, with a 2.5-mm gap maintained between each. Slice thickness was verified using a digital caliper. The canal diameters, apically and coronally, of each slice were measured using a Nikon Eclipse MA100 stereomicroscope (Nikon Corp., Tokyo, Japan) at 25× magnification prior to performing the POBS test. Each slice was subjected to compressive load using a computer-controlled universal testing machine (Instron Corp., Massachusetts, USA). A flat-tip stainless steel cylindrical plunger was used to apply the load, with diameters of 0.9 mm for the coronal third, 0.7 mm for the middle third, and 0.5 mm for the apical third. These sizes were chosen in accordance with previous studies to ensure that the plunger diameter remained strictly within 60%–85% of the pre-measured lesser canal diameter (apical side) of each specimen, ensuring direct load execution on the obturation material without exerting stress on the surrounding dentin [20, 21]. Load was applied perpendicular to the surface of each specimen in an apico-coronal direction. Maximum load recorded was considered the POBS. The highest force at displacement was recorded in Newtons (N) and converted to megapascals (MPa) using the formula [13]:

$$\text{POBS = F/A} = \mathrm{F/}\left(\pi\times\left(\left(\mathrm{D1+D2}\right)/2\right)\times\mathrm{h}\right)$$

Where:

- F = Applied force at displacement (N)
- A = Surface area under load (mm²)
- h = Thickness of the root slice (mm)
- D1 = Greater canal diameter (coronal side)
- D2 = Lesser canal diameter (apical side)
- $$\pi$$= Constant ($$\approx$$ 3.14)

### Failure mode analysis

Failure modes were assessed at 40× magnification using a stereomicroscope and classified as: cohesive failure (CF), occurring within the sealer with sealer remaining on dentin; adhesive failure (AF), characterized by complete detachment from dentin with no sealer residue; and mixed failure (MF), a combination of CF and AF with partial sealer retention on canal walls.

### Statistical analysis

Data were statistically analyzed using GraphPad Prism 10.0 (GraphPad Software, CA, USA). Data normality was assessed using the Shapiro–Wilk test, and homogeneity of variances was evaluated using Brown–Forsythe test. POBS findings are presented as mean ± standard deviation (SD). Three-way ANOVA followed by Tukey’s post hoc test was used to assess POBS findings. Although variance homogeneity was violated (Brown–Forsythe test: F = 2.45, *P* < 0.05), three-way ANOVA was considered appropriate and robust because the experimental design was balanced (*n* = 10 per subgroup) [22, 23]. Pooled POBS data were assessed using one-way ANOVA followed by Tukey’s post hoc test. Chi-square test was used to analyze distribution of failure modes. The level of significance was set at (*P* < 0.05).

## Results

### Characterization of NC

#### TEM analysis

**Fig. 2:**
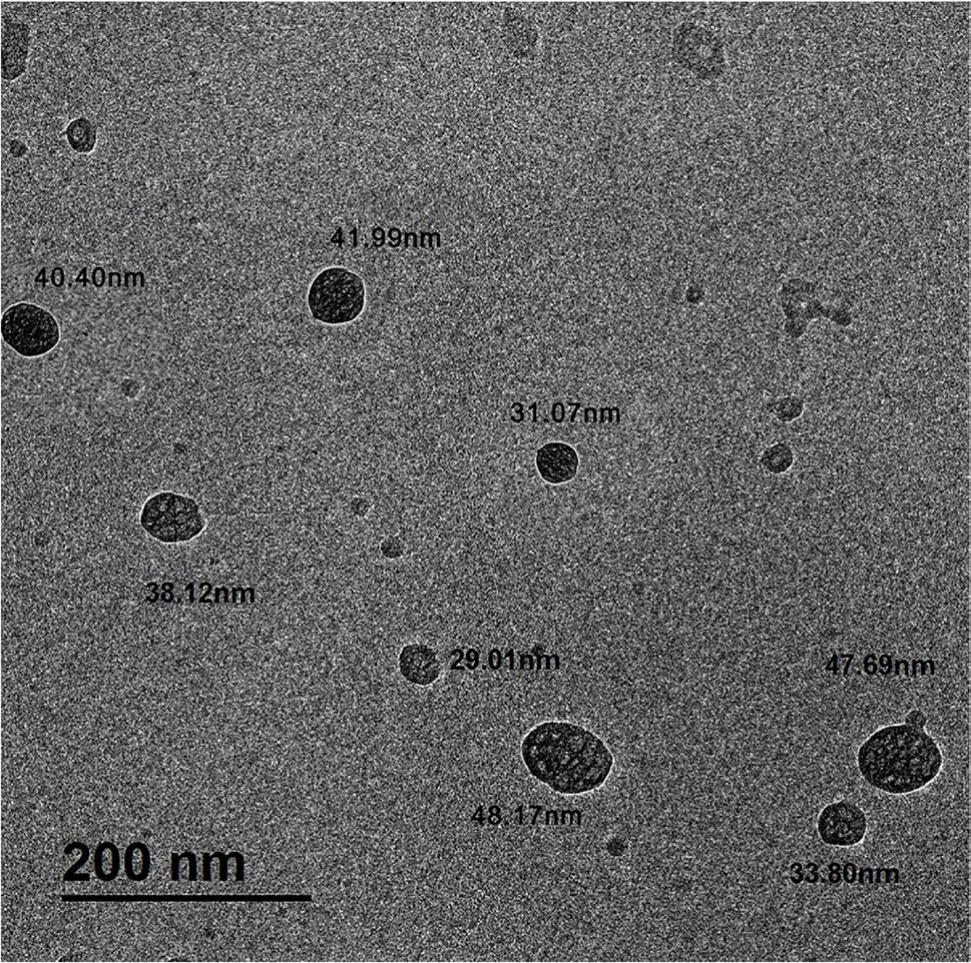
TEM analysis of nano-chitosan

TEM analysis confirmed the nanoscale dimensions of the particles, which appeared mostly spherical but with some irregular and undefined shapes, thereby increasing their effective surface area. Individual particles were observed within the nanometer range, with measured diameters less than 50 nm. The analysis also indicated that the nanoparticles remained relatively well-dispersed in solution (Fig. 2).

#### DLS and zeta potential measurement

DLS analysis of NC revealed a hydrodynamic Z-average diameter of 62.4 nm. The size distribution profile displayed a single sharp peak with a mean by intensity of 65.1 nm. The polydispersity index was found to be 0.195, confirming a narrow, monodisperse distribution of the particles in the aqueous phase. The results have showed that NC carries a positive charge, with an average zeta potential of + 47.57 mV, indicating good colloidal stability and strong potential for electrostatic interactions.

### Findings of POBS

#### Three-way ANOVA findings

Table 1 presents the results of the three-way ANOVA assessing the effects of intracanal medication, sealer type, and root level, and their interactions on POBS. POBS was significantly influenced by the “medication” factor (*P* < 0.05). However, neither the “sealer” nor the “root level” factors had significant impact on POBS (*P* > 0.05). POBS was significantly impacted by “medication/sealer”, “medication/root level” and “sealer/root level” interactions (*P* < 0.05). No significant three-way interaction was found among the “medication”, “sealer”, and “root level” factors (*P* > 0.05).

**Table 1:** Three-way ANOVA showing the effects of intracanal medication, sealer type, and root level, as well as their interactions on POBS

Variation Factors	df	Mean Square	F		ƞ_*p*_^2^	*P*-value
Medication	2	41.7747	60.0455		0.426	< 0.001*
Sealer	1	0.0679	0.0976		0.001	0.755
Root level	2	1.0188	1.4644		0.018	0.234
Medication * Sealer	2	2.4504	3.5221		0.042	0.032*
Medication * Root level	4	9.7913	14.0737		0.258	< 0.001*
Sealer * Root level	2	10.8396	15.5805		0.161	< 0.001*
Medication * Sealer * Root level	4	1.0111	1.4533		0.035	0.219
Residuals	162	0.6957				

* Statistically significant difference at *P* < 0.05

*df* degrees of freedom, *F* Fisher’s F -statistic, ƞ_p_^2^ partial eta squared

#### Effect of intracanal medication on POBS of each sealer at different root levels

#### Regarding POBS of AH Plus

At coronal level, there was no significant difference among the subgroups IA, IIA and IIIA (*P* > 0.05). At middle level, subgroup IA showed significantly higher POBS than subgroups IIA and IIIA (*P* < 0.05). At apical level, POBS of subgroup IA was significantly greater than that of subgroup IIIA, which in turn exhibited significantly higher bond strength than that of subgroup IIA (*P* < 0.05) (Table 2).

**Table 2:** Mean ± SD of POBS [MPa] of the different subgroups at each root level

Root level	Medication	Control (I)	CH (II)	CH/NC (III)	*P*-value
	Sealer				
Coronal	AHP	2.971 ± 1.556	2.889 ± 0.3421	3.221 ± 0.6516	0.741
	NSF	2.659 ± 0.3702^A^	2.084 ± 0.8207^AB^	1.518 ± 0.8023^B^	0.0043*
	*P*-value	0.5447	0.0103*	< 0.0001*	
Middle	AHP	3.408 ± 0.3358^A^	1.647 ± 0.7807^B^	2.083 ± 1.037^B^	< 0.0001*
	NSF	3.51 ± 1.271^A^	2.702 ± 0.829^AB^	2.326 ± 0.4561^B^	0.0226*
	*P*-value	0.8097	0.0089*	0.5064	
Apical	AHP	4.322 ± 0.9489^A^	1.022 ± 0.5575^C^	2.187 ± 0.9403^B^	< 0.0001*
	NSF	4.799 ± 0.8595^A^	2.019 ± 0.7822^B^	2.483 ± 0.5923^B^	< 0.0001*
	*P*-value	0.2543	0.0041*	0.4102	

* Statistically significant difference at *P* < 0.05

Different superscripted uppercase letters indicate significantly different values within the same row (*P* < 0.05)

#### Regarding POBS of NeoSEALER Flo

At coronal and middle levels, subgroup IB exhibited significantly higher POBS compared to subgroup IIIB (*P* < 0.05). However, subgroup IIB did not show significant difference from either subgroup IB or subgroup IIIB (*P* > 0.05). At apical level, subgroup IB showed significantly higher POBS than both subgroups IIB and IIIB (*P* < 0.05) (Table 2).

#### Comparison between AHP and NSF sealers within each group

For control group, regardless of the root level, no significant difference was observed between AHP and NSF (*P* > 0.05). For CH group, at coronal root level, AHP was significantly superior to NSF (*P* < 0.05). Conversely, at middle and apical root levels, AHP was significantly inferior to NSF (*P* < 0.05). For CH/NC group, at coronal root level, POBS of AHP was significantly higher than NSF (*P* < 0.05). Meanwhile, no significant difference was noted between AHP and NSF at middle and apical root levels (*P* > 0.05) (Table 2).

#### Pooled data of POBS

Pooled POBS data revealed that the application of medicaments led to a significant reduction in bond strength (*P* < 0.05). Medication subgroups (IIA, IIB, IIIA, and IIIB) exhibited significantly lower values, ranging from 1.853 to 2.497 MPa, compared to 3.567 to 3.656 MPa in the control subgroups (IA and IB). This represented an approximate reduction of 30%–48% relative to the control subgroups. Notably, no significant differences were found among the medication subgroups (*P* > 0.05) (Table 3).

**Table 3:** POBS pooled data [MPa] of subgroups regardless of root levels (*n* = 180)

Subgroup	Mean ± SD	*P*-value
IA (*n* = 30)	3.567 ± 1.181^A^	< 0.0001*
IB (*n* = 30)	3.656 ± 1.255^A^	
IIA (*n* = 30)	1.853 ± 0.9722^B^	
IIB (*n* = 30)	2.268 ± 0.8426^B^	
IIIA (*n* = 30)	2.497 ± 1.007^B^	
IIIB (*n* = 30)	2.109 ± 0.747^B^	

* Statistically significant difference at *P* < 0.05

Different superscripted uppercase letters indicate significantly different values within the same column (*P* < 0.05)

No significant difference was detected between the mean of pooled POBS findings of AHP (2.64 ± 1.26) and NSF (2.68 ± 1.19) (*P* > 0.05). Additionally, the mean total POBS did not differ significantly among the coronal, middle, and apical root levels, with values of 2.56 ± 1.01, 2.61 ± 1.06, and 2.81 ± 1.54, respectively (*P* > 0.05).

#### Failure mode analysis

**Fig. 3:**
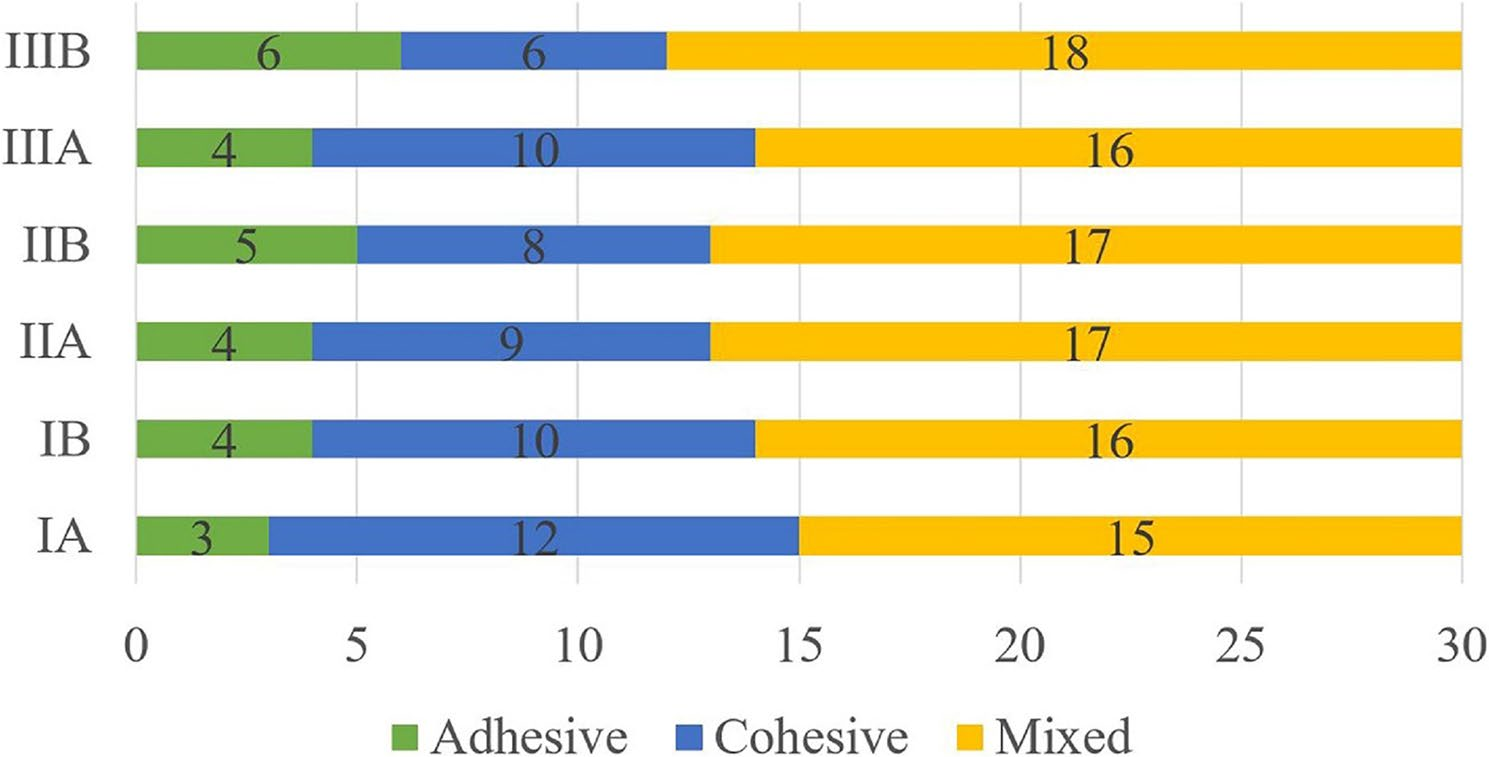
Bar chart showing the distribution of failure modes

Distribution of failure modes in root slices after POBS testing is presented in (Fig. 3). Failure mode analysis revealed that all subgroups predominantly exhibited MF, followed by CF and AF. However, there was no significant difference among tested subgroups in terms of failure mode (*P* > 0.05).

## Discussion

Adhesion is a crucial property of sealers that ensures a fluid-tight seal to prevent microleakage, supporting long-term success of endodontic treatment. It is influenced by factors such as chemical composition of sealer, setting mechanism, interaction with dentin, and condition of dentinal substrate at time of obturation [8, 9]. The quality of the dentinal surface affects adhesion because irrigants and intracanal medicaments may alter chemical and structural properties of dentin. Moreover, residual medicaments may interact with the sealer, modifying its physical or chemical traits. These changes may affect sealer flowability and setting time, impacting bond strength to root canal dentin [14, 24]. The magnitude of these effects depends on the medicament type, application time, and removal efficacy before obturation [24].

Despite their impact on dentin and sealer bonding, intracanal medicaments are essential in root canal treatment to improve disinfection, prevent reinfection of canal between visits and aid drying of weeping canals [2, 3]. CH remains the most widely used intracanal medicament, owing to its extensively proven antibacterial effectiveness against most strains typically present in root canal infections. However, its limited antimicrobial activity against *E. faecalis* and *Candida albicans* highlights the need to explore alternative medicaments or additives [3].

NC was incorporated with CH as an intracanal medicament in this study due to its synergistic benefits with CH for root canal disinfection and its potential adhesion enhancing ability [7, 15]. NC supports sustained ion release over time and enhances antibacterial effectiveness especially against resistant microorganisms like *E. faecalis* [7]. According to Harishma et al. [15], incorporation of NC into ERBSs and CSBSs enhanced their bond strength to root canal dentin compared to unmodified sealers.

Bonding ability of endodontic sealers throughout different root levels may vary due to anatomical differences such as density of dentinal tubules and permeability of dentin [5]. To our knowledge, no data are available regarding the effect of incorporating NC to CH as intracanal medicament on POBS of AHP or NSF sealers. Moreover, existing data on the effect of CH on POBS of AHP and bioceramics are conflicting [25–29]. Some studies reported that residual CH reduced sealer adhesion and bond strength to root canal dentin [13, 27, 29]. In contrast, other studies found that prior application of CH significantly enhanced sealer POBS [26, 30], while others observed no significant effect [25, 28, 31]. Therefore, this study was conducted to assess the impact of CH combined with NC as an intracanal medicament on POBS of AHP and NSF at different root levels, compared to the commonly used CH.

Specimen standardization plays a vital role in endodontic investigations. In this study, maxillary central incisors with uniform root lengths were chosen and prepared using X5 file (50/0.06) to ensure consistent dimensions for subsequent mechanical testing. This was to provide standardized, tapered canal geometries with cross-sectional diameters that closely matched those of the universal testing machine plungers, thereby facilitating accurate and reliable POBS testing [32]. ProTaper Next system shows adequate shaping ability and maintains canal anatomy due to its off-centered rectangular cross-section creating an asymmetric rotary motion. Its unique swaggering movement within root canal enhances activation of irrigating solution during shaping process [19].

To further minimize the influence of anatomical variability on POBS measurements, canal diameters were measured at each slice prior to POBS testing, and plunger sizes were selected individually to maintain a consistent ratio (60%–85% of the apical diameter, D2) [20, 21]. This approach ensured standardized stress application across specimens while accounting for inherent differences in canal morphology.

Characterization of NC was performed using TEM, DLS and zeta potential measurement. TEM micrographs indicated that nanoparticles were predominantly spherical, although some exhibited irregular and less defined shapes, with diameters less than 50 nm. Moreover, NC particles dissolved in glacial acetic acid were well dispersed. DLS analysis yielded a hydrodynamic Z-average diameter of 62.4 nm with polydispersity index value of 0.195, indicating a relatively narrow size distribution and good dispersion of the nanoparticles in the aqueous phase. The larger particle size measured by DLS compared with TEM is expected because TEM measures the physical diameter of dehydrated nanoparticles, whereas DLS determines the hydrodynamic diameter of particles in suspension, including the surrounding hydration layer and electrical double layer. Zeta potential measurement showed a positive surface charge of + 47.57 mV, which suggests good colloidal stability and a strong potential for electrostatic attraction with negatively charged dentin surfaces. This positive charge may also improve antibacterial effectiveness of NC by promoting stronger adherence to bacterial cell walls through electrostatic interactions, potentially enhancing its function as an intracanal medicament [33–35].

Powder form of CH was chosen to avoid concerns related to oily or viscous vehicles in commercial paste formulations that may hinder dissociation and release of hydroxyl ions [36]. Additionally, certain additives like barium sulfate may theoretically compromise bond strength due to their challenging removal [37]. For CH/NC, glacial acetic acid was used to dissolve NC before mixing CH with it; overcoming the concern of NC powder aggregation [4].

Intracanal medicament removal was achieved using 17% EDTA with PUI. Rödig et al. [38] and Ghabraei et al. [13] demonstrated that chelating agents combined with PUI eliminated CH residues more effectively than NaOCl. Superior performance of EDTA may be attributed to its ability to disrupt and dissolve CH remnants. Additionally, PUI has been shown to outperform manual hand filing in eliminating CH remnants, particularly in root canals with complex anatomy, where traditional mechanical techniques may have limited effectiveness in accessing and thoroughly cleaning irregular canal surfaces [13].

This study employed the POBS test to measure bond strength between root canal sealers and dentin. This method is well-regarded for simulating clinical shear stresses at dentin–sealer interface [21]. It offers precise specimen standardization and produces reliable results, even when bond strengths are low. Unlike tensile and shear tests, the POBS test is less affected by differences in specimen shape and stress distribution, and it enables evaluation of bond strength at various levels of the root [39].

According to the three-way ANOVA findings, POBS was significantly influenced by the use of CH and CH/NC, as well as by the interactions between medication and sealer types, and between medication type and root level. The significant medication–sealer interaction indicates that the effect of intracanal medication was not uniform across sealer types. Although both CH and CH/NC reduced POBS relative to the corresponding control subgroups, the response pattern varied between the two sealers. Subgroup IIA exhibited the lowest pooled mean bond strength (1.853 MPa), representing the greatest reduction compared with its corresponding control subgroup IA (3.567 MPa), and therefore appeared to be the most vulnerable sealer–medicament combination. While the mean POBS values of AHP were numerically higher in the CH/NC group than in the CH group, this difference was not statistically significant. Likewise, no significant differences were detected among the medicated subgroups in the pooled analysis. These findings suggest that intracanal medicaments affected the two sealers differently; however, under the conditions of the present study, the addition of NC to CH did not provide a statistically significant improvement in bond strength compared with CH alone.

Generally, the use of medication significantly reduced POBS of both AHP and NSF compared to control group. Residues of medication may act as a physical barrier between the sealer and the root dentin [14]. Additionally, these remnants may chemically interact with the sealer, adversely affecting its properties such as flow, working time and film thickness [24]. These alterations may reduce the bond strength of the sealer to dentin and ultimately compromise the apical seal [14, 24].

Regarding the findings of the compromising effect of CH on POBS of AHP, they came in line with previous reports by Guiotti et al. [14], Dewi et al. [27] and Sahebi et al. [5]. Residual CH may have interfered with bonding of AHP to root dentin by hindering its chemical interaction, reducing covalent bond formation, and limiting resin penetration into dentinal tubules [14, 40]. On the other hand, Carvalho et al. [30] and Maan et al. [26] reported that CH enhanced POBS of AHP. This inconsistency may be attributed to variations in experimental protocols, such as canal irrigation method, CH removal method or obturation technique.

Differences in ease of medicament removal across root canal, with the apical level being the most challenging, contribute to the regional variation in bond strength as reported by Üstün et al. [31]. Notably in this study, at apical level, POBS of AHP in the CH/NC group was significantly higher than that in the CH group. This suggests that incorporating NC to CH may reduce the adverse effect of CH on bond strength of AHP at this level. It is noteworthy that addition of NC to sealer potentially improves bond strength; as NC reinforces dentin collagen by increasing cross-linking and inhibiting matrix metalloproteinases [15, 41].

CH and CH/NC significantly lowered POBS of NSF compared to control group with no significant difference between CH and CH/NC. Taking into consideration that limited data are available regarding effect of CH on NSF, but as a bioceramic sealer, the findings of CH group came in agreement with previous reports by Ghabraei et al. [13] and Ghoneim et al. [29]. However, Amin et al. [42] and Maan et al. [26] reported that CH enhanced POBS of bioceramics sealers. The different findings may stem from variations in the study design such as the form of CH, incubation period, CH removal method, obturation technique or type of used bioceramic sealer.

 Harishma et al. [15] reported that direct incorporation of NC into an ERBS and a CSBS enhanced their bond strength to root dentin compared with the corresponding unmodified sealers. In contrast, the pooled data of the present study showed that CH/NC intracanal medicament did not show statistically significant improvement POBS compared with CH alone, and both medicaments reduced bond strength relative to the unmedicated controls. This discrepancy may be related to the site of NC application. When incorporated into the sealer, NC becomes part of the sealer matrix and may strengthen the adhesive interface through interaction with dentinal collagen. Conversely, when applied as an intracanal medicament, its potential beneficial effects may be outweighed by the presence of residual CH/NC remnants on the dentinal surface that may interfere with sealer–dentin bonding by acting as a physical barrier. Therefore, the effect of NC on adhesion appears to be highly dependent on whether it is incorporated into the sealer or applied as an intracanal medicament.

Pooled data of the present study revealed that sealer type did not significantly affect POBS as AHP and NSF showed comparable bond strength to root canal dentin. AHP achieves adhesion to dentin chemically by forming covalent bonds between its epoxy rings and collagen amino groups of dentin, and mechanically through monomer penetration that creates a hybrid layer of polymerized resin intertwined with collagen fibers [10]. NSF bonds to root canal dentin by penetrating dentinal tubules for micromechanical retention and forming a chemical bond through hydroxyapatite deposition and formation of mineral infiltration zone [43, 44]. Recently, Pradelli et al. [45] and Kandil et al. [46] reported superior POBS values for NSF compared with AHP. They attributed favorable bonding performance of NSF to its bioactive calcium silicate composition and physicochemical properties, particularly its hydrophilic nature and low contact angle, which promote optimal adaptation to root canal dentin.

In this study, at middle and apical levels in CH group, POBS of AHP was significantly lower than that of NSF. According to Üstün et al. [31], medicament removal apically is the most challenging while easier when progressing towards coronal level. The findings by Guiotti et al. [14] and Tavella et al. [40] are suggestive that residual CH interferes strongly with ERBSs compared to CSBSs [40]. Guiotti et al. [14] and Tavella et al. [40] explained that CH residues may alter chemical and mechanical interaction between ERBSs and dentin by creating a barrier that inhibits proper infiltration and polymerization of epoxy resin, whereas CSBSs were less affected, as their hydration-based setting reaction required less dentin surface cleanliness.

The POBS values documented for ERBSs and CSBSs exhibit considerable variability across the literature, largely due to differences in sealer formulations, experimental conditions, specimen preparation, and testing methodologies [10, 15, 43]. In the present study, mean POBS values ranged from approximately 1.0 to 4.8 MPa, which fall within the range reported in previous studies [10, 15, 43]. In laboratory investigations, POBS values are most appropriately interpreted in a comparative rather than absolute manner. Although higher POBS values are generally desirable, there is no universally accepted minimum POBS value in MPa that guarantees clinical success. Push-out testing evaluates interfacial shear resistance under standardized conditions and does not fully replicate the complex mechanical, biological, and functional stresses encountered in-vivo [8, 42]. Accordingly, the lower POBS values observed in specific subgroups and root levels may reflect comparatively lower interfacial resistance under the conditions of the push-out test; however, their direct translation into clinical performance should be interpreted with caution and considered alongside other factors such as sealer properties, failure patterns, long-term sealing ability, and overall treatment outcomes.

Chi-square test revealed no statistically significant differences in failure mode distribution among tested subgroups. Nevertheless, MF was the most predominant mode across all subgroups, suggesting that debonding was not confined to a single interface but involved both interfacial separation and cohesive disruption within the sealer. This pattern indicates relatively stable adhesion between the sealer and radicular dentin, even though the bond strength values were lower than those of the control. Similar findings were reported by Al-Hiyasat et al. [21] and Falakaloglu et al. [32] who observed a predominance of MF for AHP and bioceramic sealers. Therefore, the predominance of MF may reflect partial preservation of the adhesive interface despite the reduction in bond strength [13, 47].

This study was conducted under ex-vivo conditions using extracted teeth, which do not fully replicate the clinical environment. The absence of periapical tissue pressure and the simplified anatomical setting may influence sealer penetration and bonding behavior; therefore, the findings should be interpreted with caution when extrapolated to in-vivo conditions. In addition, POBS was evaluated after only one week of incubation. Since CSBSs undergo a prolonged hydration and maturation process, the interfacial properties and bond strength values may change over time, warranting further investigation with longer incubation periods.

The proposed explanation that residual intracanal medicament may act as a physical barrier to sealer–dentin interaction is based on indirect evidence, as scanning electron microscopy (SEM) analysis was not performed to assess dentinal tubule cleanliness or the presence of medicament remnants. Furthermore, failure mode analysis was conducted using a stereomicroscope at 40× magnification, which provides less detailed and more subjective assessment than SEM-based evaluation.

## Conclusions

Under the specific experimental conditions of this study, prior application of CH or CH/NC reduced POBS for both AHP and NSF compared with unmedicated controls. The addition of NC to CH did not provide a statistically significant improvement in POBS compared to CH alone. Overall, AHP and NSF demonstrated comparable POBS to root canal dentin.

## Data Availability

This study has been extracted from MSc thesis conducted at Mansoura University. The data supporting the findings of this study are available from the corresponding author upon reasonable request.

## Abbreviations

AF: Adhesive failure
AHP: AH Plus
CF: Cohesive failure
CH: Calcium hydroxide
CH/NC: Calcium hydroxide/nano-chitosan
CSBSs: Calcium silicate-based sealers
DLS: Dynamic light scattering
*E. faecalis*: *Enterococcus faecalis*
ERBSs: Epoxy resin-based sealers
GP: Gutta-percha
MF: Mixed failure
NaOCl: Sodium hypochlorite
NC: Nano-chitosan
NSF: NeoSEALER Flo
POBS: Push-out bond strength
PUI: Passive ultrasonic irrigation
SEM: Scanning electron microscopy
TEM: Transmission electron microscopy
